# Supplementary material for: Sensitive Protein Detection Using Site-Specifically Oligonucleotide-Conjugated Nanobodies
Source: Anal Chem. 2022 Jul 5;94(28):10054–61. doi: 10.1021/acs.analchem.2c00584 (PMC9310004; doi:10.1021/acs.analchem.2c00584)
Supplement: Supplementary file 1 — ac2c00584_si_001.pdf [file ac2c00584_si_001.pdf]

Supplementary Information for

Title: Sensitive protein detection using site-specifically oligonucleotide-conjugated nanobodies

Rasel A. Al-Amin[†,*], Phathutshedzo M. Muthelo[†,#], Eldar Abdurakhmanov[‡,#], Cécile Vincke[ξ,§,#], Shahnaz P. Amin[Ψ], Serge Muyldermans[ξ], U. Helena Danielson[‡], Ulf Landegren[†]

[†]Department of Immunology, Genetics and Pathology, Science for Life Laboratory, Uppsala University, Box 815, SE-751 08 Uppsala, Sweden.

[‡]Department of Chemistry-BMC, Science for Life Laboratory, Uppsala University, Box 576, SE-751 23 Uppsala, Sweden.
[ξ]Laboratory of Cellular and Molecular Immunology, Vrije Universiteit Brussel, 1050 Brussels, Belgium.

[§]Myeloid Cell Immunology Lab, VIB Center for Inflammation Research, Brussels, 1050 Brussels, Belgium.

[Ψ]Capio Vårdcentral Väsby, Dragonvägen 92, 194 33 Upplands Väsby, Sweden.

[#]These authors contributed equally.

Correspondence Author

***Rasel A. Al-Amin -** Department of Immunology, Genetics and Pathology, Science for Life Laboratory, Uppsala University, Box 815, SE-751 08 Uppsala, Sweden; orcid.org/0000-0002-0762-9034; E-mail: drrasel.alamin@gmail.com

Table of Contents

Material & Methods		Page
**Materials**	Materials and reagents	S3
**Method S1.**	Expression, identification and selection of IL6-specific Nbs	S3
**Method S2.**	NbIL6s clones' selection via enzyme-linked immunosorbent assays	S3
**Method S3.**	Expression, isolation and affinity measurement of NbSORIL6	S4

Tables and Figures

**Table S1.**	Summary of Nbs and tag sequences	S5
**Table S2.**	Affinity comparison of Nbs with different tags	S5
**Table S3.**	Summary of oligos sequences composition, length (nt) and modifications of oligos conjugated to the Ab and Nb used for PEA	S6
**Table S4.**	Equilibrium dissociation constants (K_D) as determined by various models	S6
**Figure S1.**	PEA assay oligos design and Nupack software prediction of secondary structure of combinatons of oligos	S7
**Figure S2.**	Dilution ELISA of the original six Nbs clones against recombinant human IL6	S8
**Figure S3-4.**	SPR sensorgrams for interactions between NbSORIL6_15 before and after conjugation to an oligo and a IL6 directly immobilized on the chip surface.	S9-10
**Figure S5.**	SPR sensorgrams of single cycle kinetics measurenet for all four NbSORIL6 products interacting with the target IL6 antigen	S11
**Figure S6.**	Gel electrophoretic validation of unpurified products of sortase-mediated oligo conjugation reactions of four different Nbs	S12
**Figure S7.**	Comparison of the effect of dimerizing Nb probes.	S13
**Figure S8.**	Gel validation of Ab-oligos conjugation by copper free click chemistry	S14
**References**		S15

Materials and Methods

Materials and reagents.

Recombinant human IL6 protein (206-IL-010), anti-human IL6 mAb (#6708) and human IL6 affinity purified polyclonal Ab AF-206-NA were obtained from R&D System Inc (USA), human IL6 recombinant protein (Cat. No. Z03034, Genscript) and anti-human IL6 mAb 13A5 were from MABTECH. Buffers used include PEA buffer (1xPBS with 1% BSA (New England Biolabs; NEB), 100 µg/ml salmon sperm DNA (Invitrogen), 100 µg/ml goat serum (Sigma-Aldrich), and 0.05% Tween-20 (Sigma-Aldrich)), probe storage buffer (1xPBS, 0.1% BSA, 0.05% NaN3), and wash buffer (1xPBS with 0.1% BSA and 0.05% Tween-20). All buffers were prepared in-house and their pHs were calibrated to 7.2. The buffers including water, TBS, PBS were all purified with Nalgene Rapid-Flow Filters (Thermo Scientific). All enzymes, dUTPs and dNTPs were purchased from NEB. The sequence compositions and modifications of the DNA oligonucleotides (oligos) are reported in Supplementary Table 2. All oligos were purchased from integrated DNA technologies (IDT). Their concentrations were measured by UV spectroscopy using a Nanodrop ND1000 spectrophotometer and the oligos were analyzed by PAGE (15% TEB-urea gel (Invitrogen).

Methods

Method S1. Expression, identification and selection of IL6-specific Nbs (NbSORIL6s). Briefly, a dromedary (*Camelus dromedarius*, housed in the Central Veterinary Research Laboratory, Dubai, U.A.E.) was immunized six times, at weekly intervals, with 100 µg of recombinant human IL6 (kindly provided by the NNF Center for Protein Research, University of Copenhagen, Denmark as partner of the Affinomics Consortium - EU FP7 Grant Agreement 241481). Four days after the last immunization, lymphocytes were extracted from the anti-coagulated blood of the animal, and cDNA was prepared and used as a template in a two-step, nested PCR that amplified the gene fragments encoding the variable domain of the heavy-chain-only Abs. The amplified DNA fragments encoding Nbs were ligated into a pMECS phagemid vector and transformed into *Escherichia coli* TG1 cells (Lucigen), resulting into a cloned Nb-library of 7.7×10^8 transformants of which 80% contained a phagemid with the expected insert size for a Nb. After three rounds of phage selection, 24 individual clones were cultured from each round of panning, and their periplasmic proteins were tested for specificity against recombinant human IL6 protein in an ELISA.[1] The ELISA-positive clones were selected for DNA sequencing (VIB Genetic Service Facility, Antwerp, Belgium) of their phagemid insert. The inserted DNA sequences were *in silico* translated into amino acid sequences, aligned according to the international ImMunoGeneTics database (http://imgt.cines.fr, accessed on 25 October 2019), and manually annotated.[2] Six different IL6-specific Nbs (NbIL6_5,15, 16, 21, 25 and 91) were selected and transformed in *Escherichia coli* WK6 cells for periplasmic expression. Following extraction from the periplasm by osmotic shock, these 6 Nbs (containing a HA and a His-tag at the C-terminus) were purified by IMAC and SEC. NbIL6_91 produced very poorly and could only be purified in small quantities. We further performed an enzyme-linked immunosorbent assays (ELISA) to select the most suitable IL6 binders.

Method S2. NbIL6s clones' selection via enzyme-linked immunosorbent assays (ELISA). A 96-well microplate (Thermo scientific Nunc Maxisorp™) was coated with 100 µl/well of IL6 antigen (at 2 µg/ml in ELISA coating buffer (0.1 M $NaHCO_3$, pH 8.2)) and incubated overnight at 4°C. Next, the wells were rinsed 5 times using PBST20 buffer (PBS + 0.05% Tween-20),

blocked for 120 min at room temperature (RT) with 200 µl skimmed milk powder in PBS (1% w/v) and again washed 5 times using PBST20 buffer. The NbIL6-HAHis6 were then added to the wells in a 4-fold serial dilution starting from a concentration of 8 µM to 30.5 pM in PBS. After 60 min incubation at RT, the plate was washed 10 times with PBST20 buffer. Detection of attached Nb was performed with a monoclonal mouse anti-HA Ab (Biolegend; 1 µg/ml) followed by a 2000-fold diluted goat anti-mouse IgG-alkaline phosphatase conjugated Ab, each for 60 min at RT. Finally, the plate was rinsed 5 times with PBST20 and the color was developed with 4-phenylphosphate disodium substrate (Sigma-Aldrich) (2 mg/ml) and read at 405 nm after 30 min of reaction.

Method S3. Expression, isolation and affinity measurement of NbSORIL6.

Recombinant protein binding reagents may be modified by site-directed conjugation, without risking inactivation of the affinity reagent. To allow coupling of the oligos required for PEA to the Nbs via a SrtA reaction, four IL6-specific Nbs (NbSORIL6s) were recloned to include a C-terminal sortase tag (NbSORIL6s). To this end, these Nbs were cloned into the pTEVSOR expression vector, containing a TEV-cleavage site (TENLYFQS), a sortase recognition site (LPETGG), a His6-tag and a C-tag (EPEA) downstream of the Nb (summary of Nbs and tag sequences in Supplementary Table S1). The Nb genes were amplified by PCR, and *PstI* and *Eco91I* (ThermoScientific) digestion fragments were ligated into the pTEVSOR vector cut with the same restriction enzymes. After expression and purification, the affinities for IL6 of these Nbs with or without sortase tags, were measured via SPR (Supplementary Table 2). The analyses revealed no major differences in binding kinetics when comparing the Nbs in their original form with C-terminal HA-His6-tag versus the sortase tag-modified variants (Supplementary Table 2). The four different sortase-tag expressed Nb clones (NbSORIL6) investigated herein are NbSORIL6_5, NbSORIL6_15, NbSORIL6_16 and NbSORIL6_21.

Supplementary Table S1. Summary of Nbs and tag sequences.

Nbs	C-terminal tags				MW (Da)
	**TeV-tag**	**Sor-tag**	**His6-tag**	**C-tag**	
NbSORIL6_5	TENLYFQS	LPETGG	HHHHHH	EPEA	16912
NbSORIL6_15	TENLYFQS	LPETGG	HHHHHH	EPEA	15823
NbSORIL6_16	TENLYFQS	LPETGG	HHHHHH	EPEA	16444
NbSORIL6_21	TENLYFQS	LPETGG	HHHHHH	EPEA	15459

Supplementary Table S2. The affinity of the IL6-specific Nbs (NbIL6) with C-terminal HA-His6tags were compared to the corresponding sortase-tagged anti-IL6 Nbs (NbSORIL6).

Nbs (NbIL6)	K_D (M) 1:1	Nb (NbSORIL6)	K_D (M) 1:1
NbIL6_5	1.71×10^{-9}	NbSORIL6_5	2.03×10^{-9}
NbIL6_15	6.47×10^{-9}	NbSORIL6_15	8.14×10^{-9}
NbIL6_16	6.96×10^{-9}	NbSORIL6_16	7.41×10^{-9}
NbIL6_21	1.37×10^{-8}	NbSORIL6_21	1.0×10^{-8}

Supplementary Table S3. Summary of oligos sequences composition, length (nt) and modifications, designed for conjugation to the Ab and Nb used in PEA.

Oligos name, length (nt)	Sequence (5´-> 3´) and modifications	Suppliers
H1, 33	5'-phosphate-TCGTGTCTAAAGTCCGTTACCTTGATTTCTCTC-linker-3'-Gly-Gly-Gly	Biosynthesis
H2, 35	Gly-Gly-Gly-linker-5'-TTTTTTCATCGCCCTTGGACTACGACTAAATCGTG-3'	Biosynthesis
F1, 37	Gly-Gly-Gly-linker-5'-ATATACGTGATTGACCAGACCCAGTGGAGTGGGAGTC-3´	Biosynthesis
R1, 37	Gly-Gly-Gly-linker-5'-ATATAGCTCGATTCCATGAACTTTCCCGTATAAACTC-3´	Biosynthesis
HF1, 89	GAGTTTATACGGGAAAGTTCATGGAATCGAGCCGCAGTTAATGTGATATGGCCTGCACCTTATGCTACCGTGACCTGCGAATCCAGTCT	IDT
HR1, 73	GACTCCCACTCCACTGGGTCTGGTCAATCACGCACGACTCTAGCATGTCTACGTCACGATGAGACTGGATGAA	IDT
DimerS1-F, 111	AGTTTATACGGGAAAGTTCATGGAATCGAGCCCATTCAGTCCGATGCCTCTTACCTCCCGCACGTTAAGCCATGACTCTAGCATGTCTACGTCACGATGAGACTGGATGAA	IDT
DimerS1-R, 121	GAGTTTATACGGGAAAGTTCATGGAATCGAGCCCATTCAGTCCGATGCCTCTTACCTCCCGCACGTTAAGCCATGACTCTAGCATGTCTACGTCACGATGAGACTGGATGAA	IDT
DimerS2-F, 121	GACTCCCACTCCACTGGGTCTGGTCAATCACGCCATTCAGTCCGATGCCTCTTACCTCCCGCACGTTAAGCCATGTGATATGGCCTGCACCTTATGCTACCGTGACCTGCGAATCCAGTCT	IDT
DimerS2-R, 119	GAGTTTATACGGGAAAGTTCATGGAATCGAGCATTAACAGACGTGACAATGGCTTAACGTGCGGGAGGTAAGGTGATATGGCCTGCACCTTATGCTACCGTGACCTGCGAATCCAGTCT	IDT
Univ Fwd (FEP), 22	CCACTGGGUCTGGTCAAUCACG	IDT
Univ Rev (REP), 22	GGGAAAGTUCATGGAAUCGAGC	IDT
F qPCR primer, 21	CTCTAGCATGTCTACGTCACG	IDT
R qPCR primer, 21	GATATGGCCTGCACCTTATGC	IDT
F qPCR primer, 22	CACGACTCTAGCATG TCTACG	IDT
R qPCR primer, 22	CGCAGTTAATGT GATATGGCC	IDT
TaqMan probes, 17	FAM-5'-TAGGTCAGAGTAGCACT-3'-MGB	IDT

Supplementary Table S4. The corresponding equilibrium dissociation constants (K_D) determined by various models are presented below:

Nbs	K_D (M) 1:1	K_D (M) Steady-State
NbSORIL6_5	1.4×10^{-9}	2.5×10^{-9}
NbSORIL6_15	1.9×10^{-9}	9.6×10^{-8}
NbSORIL6_16	6.6×10^{-9}	1.9×10^{-9}
NbSORIL6_21	1.4×10^{-9}	1.8×10^{-7}

Figure S1.

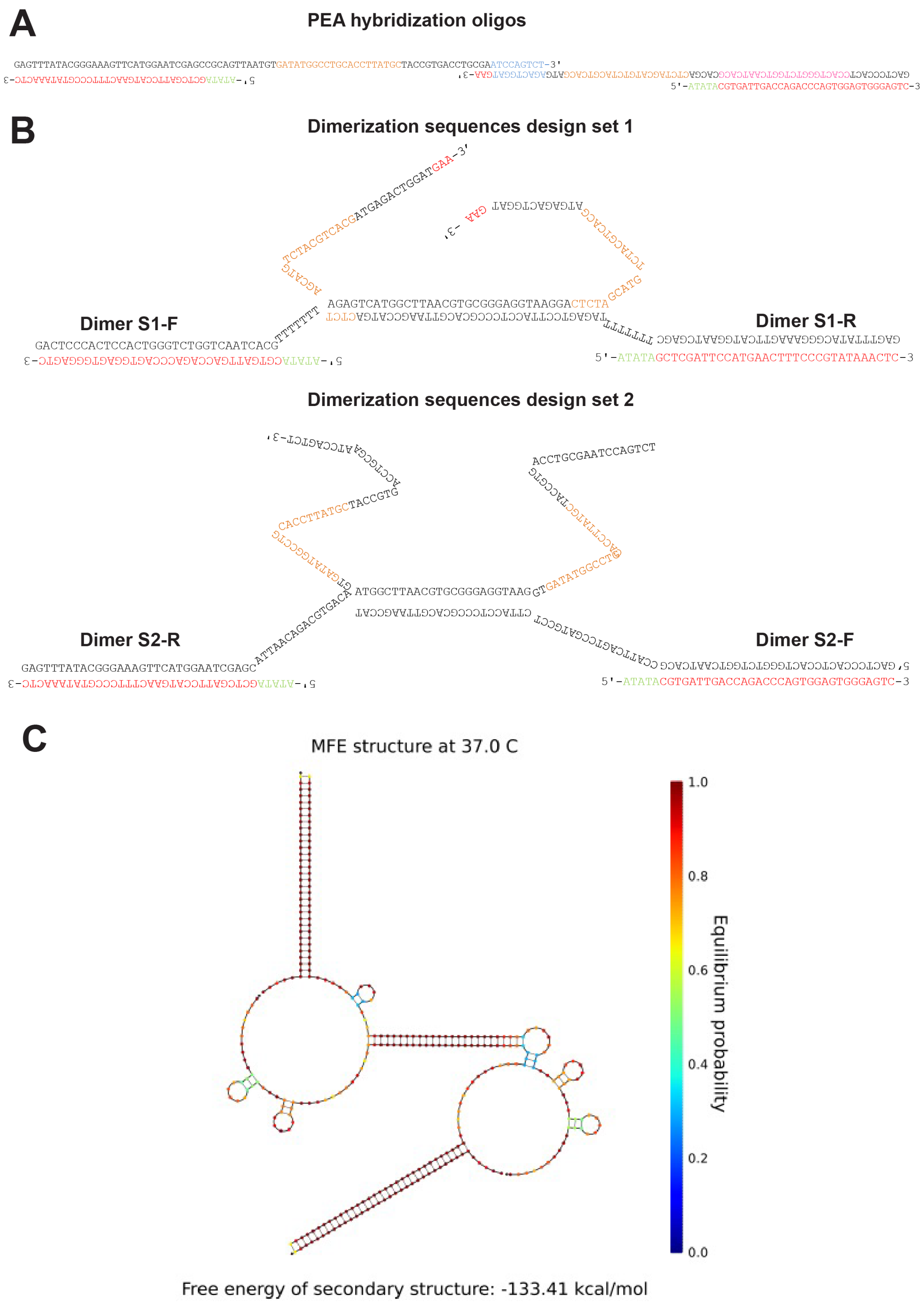

Supplementary Figure S1. (A) Design of oligos for PEA probes. **(B)** Dimerization of oligo sequences. **(C)** Nupack software prediction of secondary structure at 37°C of dimerization oligos used to form Nb homodimers through secondary hybridization (http://www.nupack.org/partition/new_from_job/3521912?token=BHLxRYMgrp).

Figure S2.

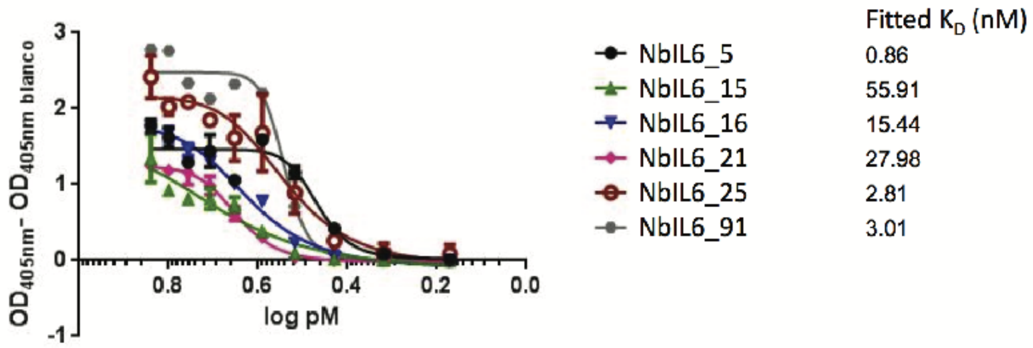

Supplementary Figure S2. The affinities of the six IL6-specific Nbs were estimated via enzyme-linked immunosorbent assays (ELISA). ELISA was performed to test the capacity of wild-type NbIL6 (containing HA and His6 tags) to bind its cognate IL6 antigen. The affinities (K_D) of the six IL6-specific Nbs for recombinant human IL6 were estimated as fllows: NbIL6_5 (0.86 nM), NbIL6_15 (55.91 nM), NbIL6_16 (15.44 nM), NbIL6_21 (27.98 nM), NbIL6_25 (2.81) and NbIL6_95 (3.01 nM).

Figure S3.

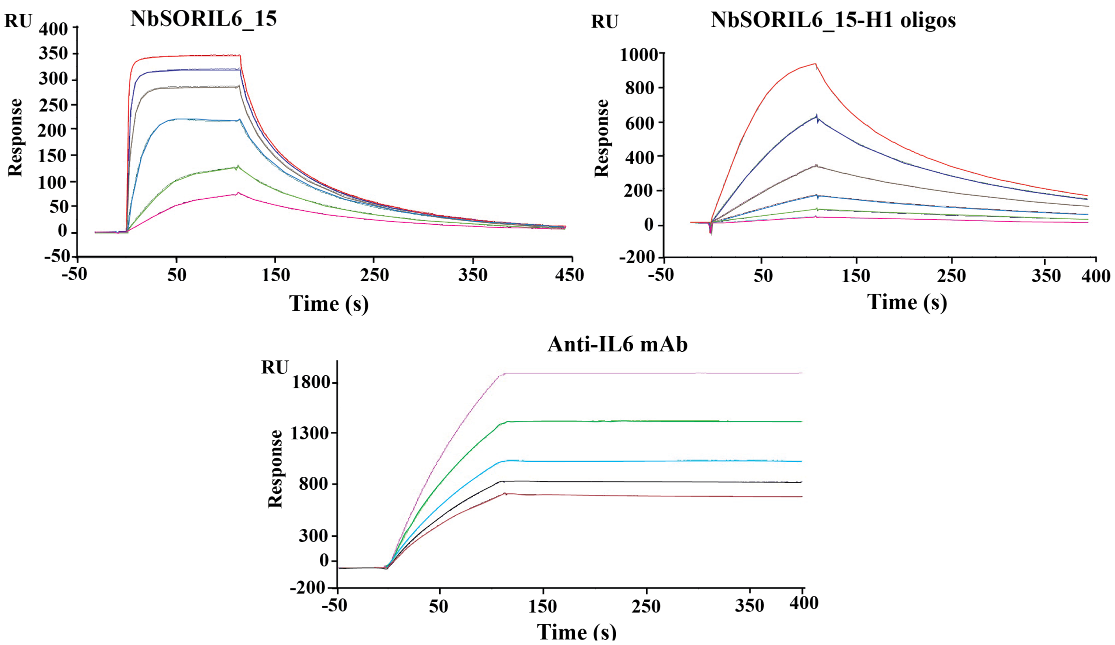

Supplementary Figure S3. Sensorgrams displaying interactions of NbSORIL6_15 with or without conjugated oligos and of an anti-IL6 monoclonal Ab (anti-IL6 mAb, clone 13A5) with IL6 immobilized directly to the chip surface. The NbSORIL6_15 and Nb SORIL6_15-oligo conjugates and the anti-IL6 mAb were injected over the IL6-coated chip surface in a series of concentrations ranging from 15.6 nM to 250 nM. The anti-IL6 mAb binds much stronger than NbSORIL6_15 and NbSORIL6_15-oligo conjugates tested with almost no observable dissociation rate from the target IL6 protein, preventing an accurate quantification of its affinity.

Figure S4.

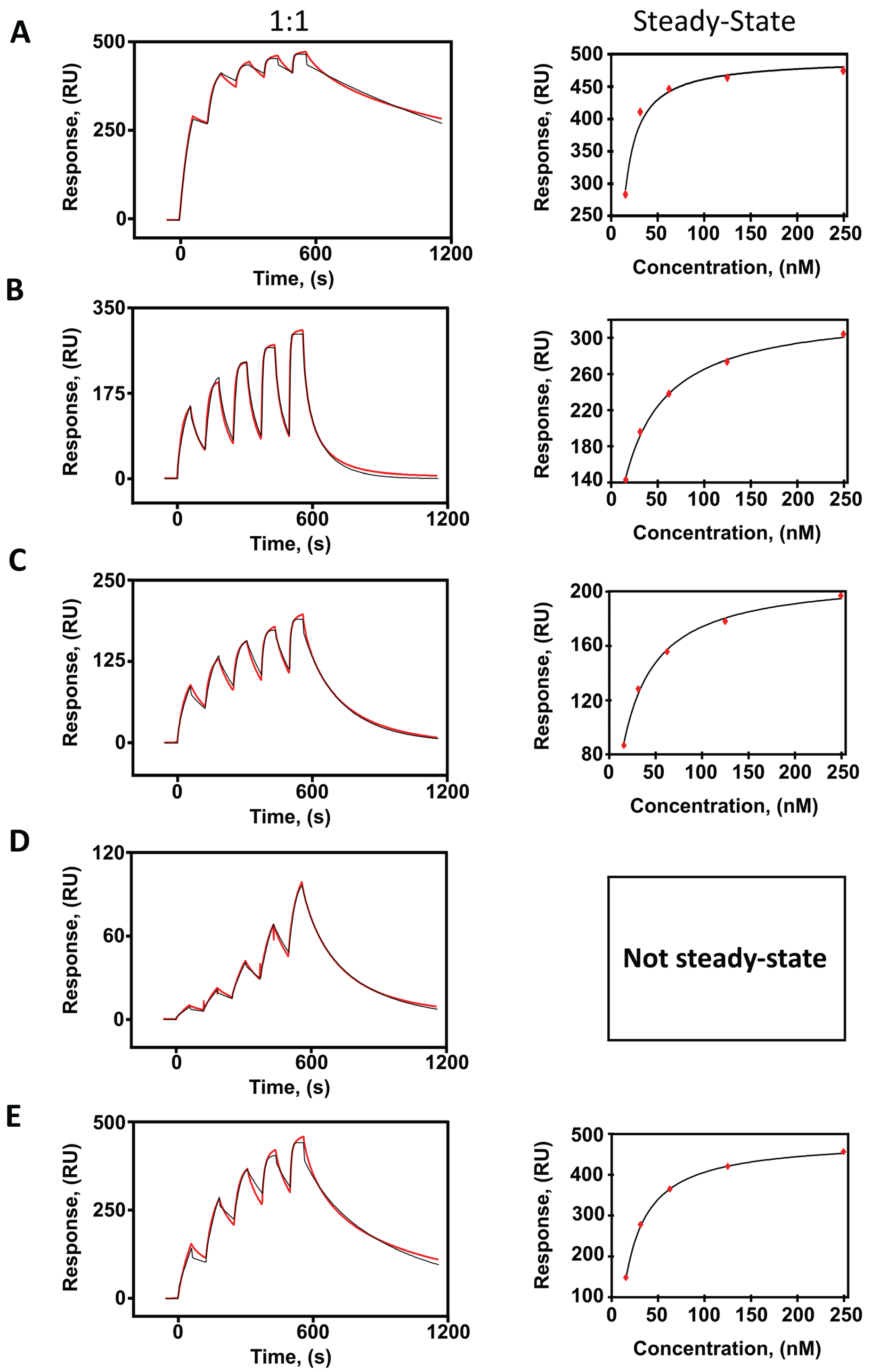

Supplementary Figure S4. SPR biosensor analysis via single cycle kinetics of NbSORIL6 and NbSORIL6-oligo conjugates interacting with IL6 immobilized directly to the chip surface. SPR sensorgrams for Nb-oligo conjugates injected over the surface in a series of concentrations from 15.6 nM to 250 nM **(A)** NbSORIL6_5, **(B)** NbSORIL6_15, **(C)** NbSORIL6_21, **(D)** NbSORIL6_21-oligos conjugates, and **(E)** NbSORIL6_16. The sensorgrams for the Nbs (black lines) were analysed by non-linear regression using a 1:1 interaction model and a steady-state. The corresponding equilibrium dissociation constants (K_D) determined by various models are presented in Table 1.

Figure S5.

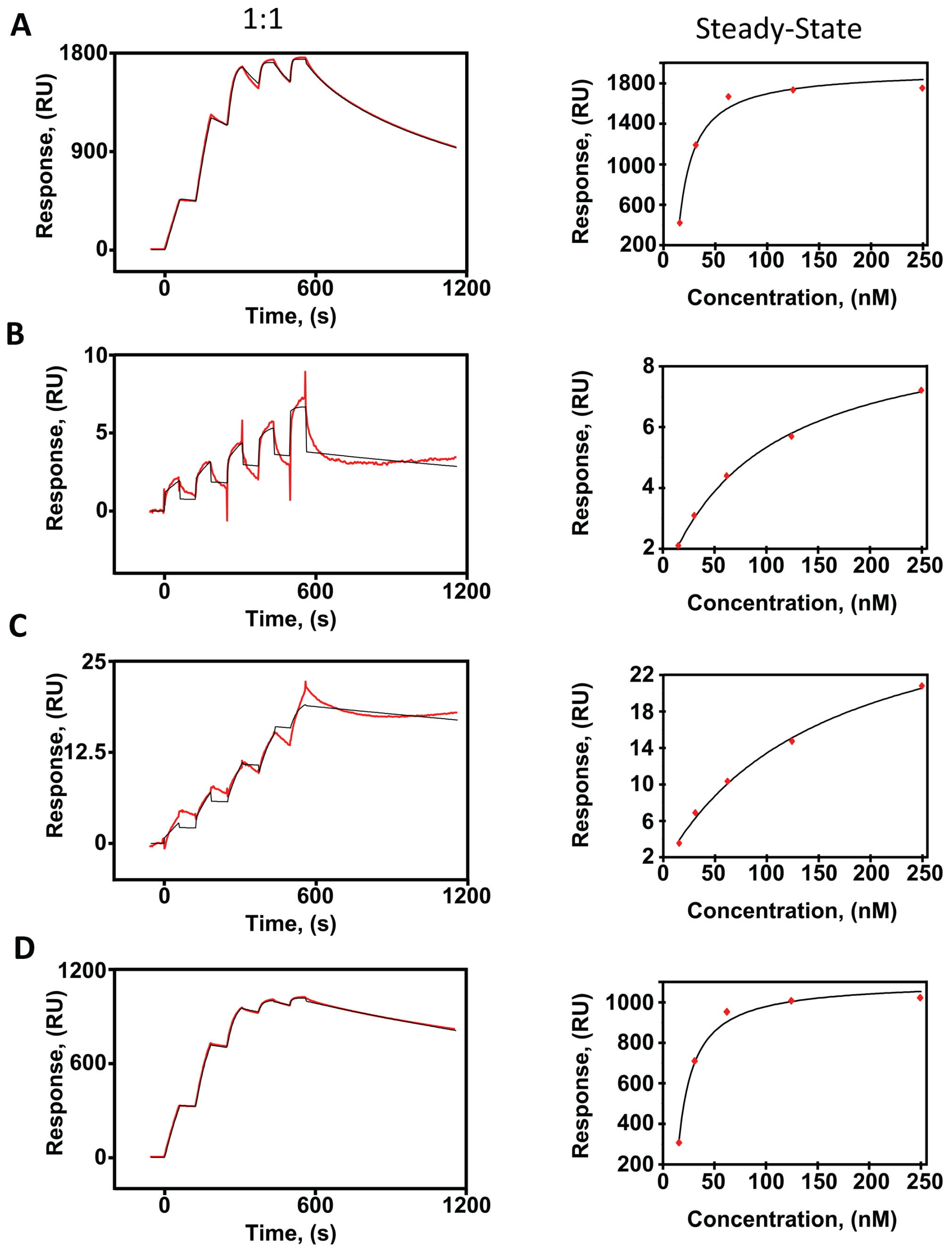

Supplementary Figure S5. Interactions between Nbs and IL6 captured on mAb that has been immobilized on the chip surface. Since the NbSORIL6 showed a slow dissociation from the target protein, a single-cycle kinetics experiment was performed. The sensorgram results, shown in red, were fitted to the different interaction models in black. The data were fitted to 1:1 and steady-state affinity models. The left column represents fitting to a 1:1 interaction mode and the right represents a steady state analysis. For all four NbSORIL6 SPR sensorgrams representing the interaction between **(A)** NbSORIL6_5, **(B)** NbSORIL6_15, **(C)** NbSORIL6_21 and **(D)** NbSORIL6_16 to the target IL6 antigen.

Figure S6.

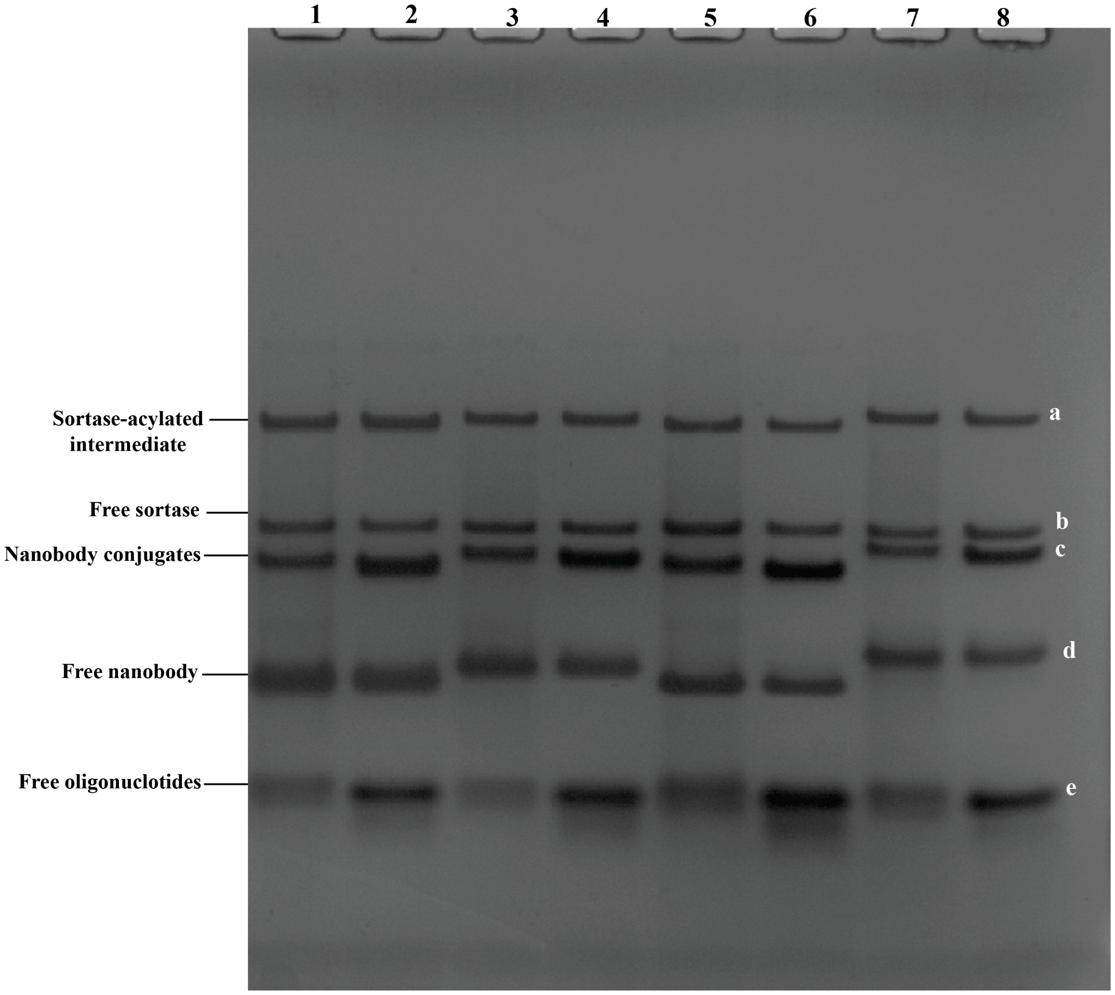

Supplementary Figure S6. Gel electrophoresis validation of unpurified sortase-mediated conjugation reactions of four different Nbs (NbSORIL6_21 16, 15 and 5) with a 5-fold molar excess of NbSORIL6 over two different oligos (H2 and H1). The species identified on the gel in order of increasing migration were: a) acylated intermediate b) SrtA enzyme c) conjugates d) excess Nb (NbSORIL6) and e) free oligos (H2 & H1). Unpurified reaction products for lanes 1 and 2: NbSORIL6_21-oligos H2 and H1, lanes 3 and 4: NbSORIL6_16-oligos H2 and H1, lane 5 and 6: NbSORIL6_15-oligos H2 and H1, lanes 7 and 8: NbSORIL6_5-oligos H2 and H1.

Figure S7.

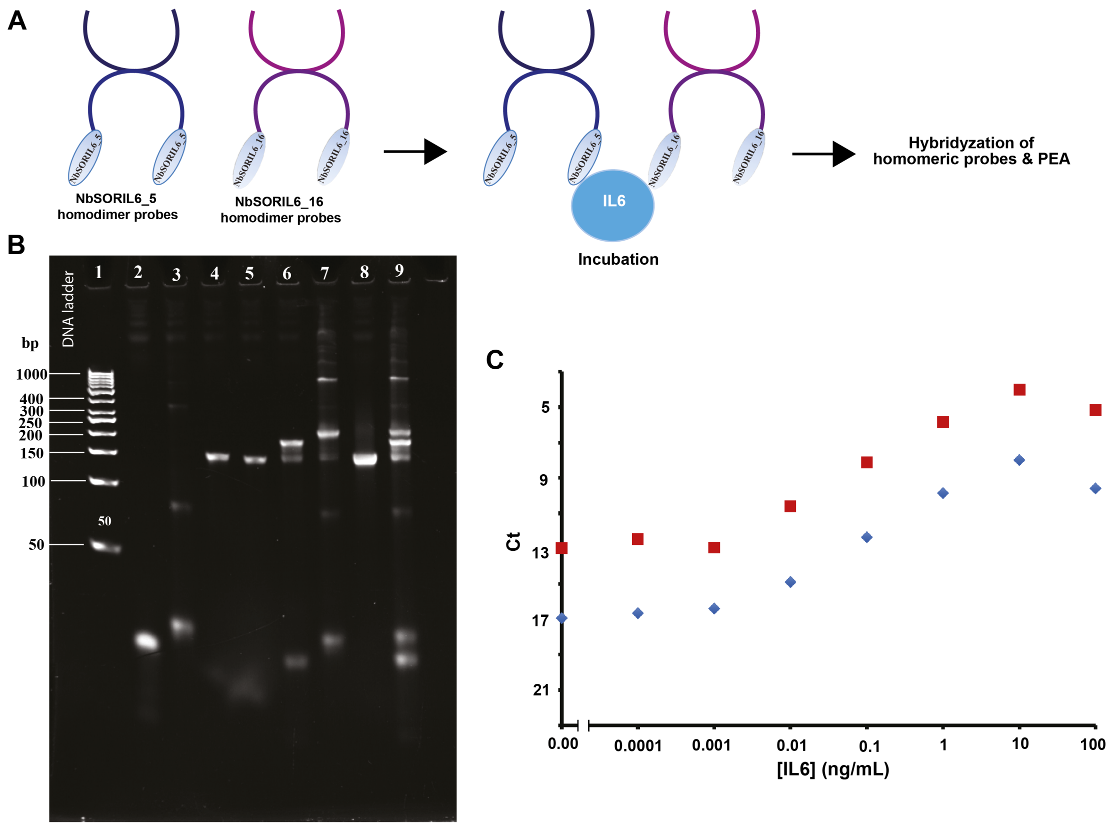

Supplementary Figure S7. Comparison of the effect of dimerizing Nb probes. **(A)** Illustration of homo-dimerized Nbs in PEA assays for IL6 protein detection. Upon incubation with IL6, homodimeicr pairs of Nb probes (NbSORIL6_5 and _16) bound to antigen were brought in proximity, allowing their attached oligos to hybridize and combine via DNA polymerization reactions. The resulting DNA extension products were quantified by real-time PCR as a measure of the amount of IL6 antigen in the sample. **(B)** Evaluation of reagents by denaturing (?) polyacrylamide gel electrophoresis (PAGE); 50-bp DNA size marker (lane 1), lanes 2 and 3 for primary GGG-modified conjugation oligos, secondary hybridization oligos (lanes 4 and 5), hybridized primary and secondary oligos (lanes 6 and 7), dimerization of the secondary oligos formed a circularized padlock probe (lane 8) and all four oligos (lane 9). **(C)** Comparison of IL6 detection by PEA using homodimerized Nb probes or monomeric NbSORIL6_5 and NbSORIL6_16 probes. PEA results of monomeric NbSORIL6_5 and _16 are indicated with a blue line while the red line corresponds to results for the homo-dimeric pairs of Nb probes.

Figure S8.

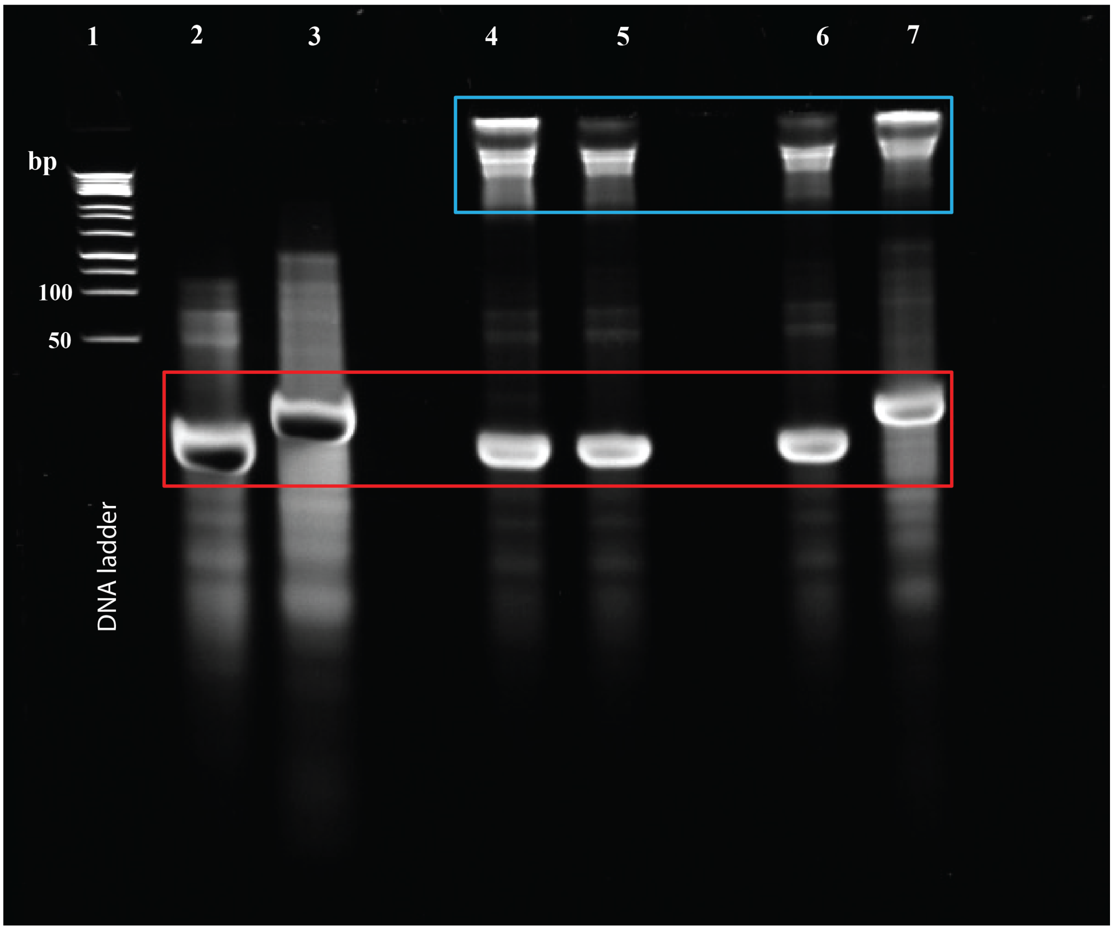

Supplementary Figure S8. Gel validation of Ab-oligos conjugation by copper free click chemistry. Ab-oligo conjugates were validated by agarose gel electrophoresis, bands in the red box in lanes 2, 3, 4, 5, 6 and 7 are free oligos while the Ab-ssDNA conjugates are in the blue box in lanes 4, 5, 6 and 7. In contrast to site-directed Nb-oligo conjugates, Ab-oligo conjugation produces multiple species with varying number of oligos conjugated to the individual Abs at different locations.

References

(1) Vincke, C.; Gutiérrez, C.; Wernery, U.; Devoogdt, N.; Hassanzadeh-Ghassabeh, G.; Muyldermans, S. Generation of single domain antibody fragments derived from camelids and generation of manifold constructs. *Methods Mol. Biol.* **2012,** *907,* 145–176.

(2) Lefranc M. P. IMGT, the international ImMunoGeneTics database. *Nucleic Acids Res.* **2003,** *31,* 307–310.
